# Multiple Abdominal Wall Chronic Discharging Sinuses With Prolene Granuloma: A Case Report

**DOI:** 10.7759/cureus.69915

**Published:** 2024-09-22

**Authors:** Ganugapanta Prem Sai Reddy, Samir Ahmad, Srinivasan C, Evangeline P Christina, Karpagam R K

**Affiliations:** 1 Department of General Surgery, Sree Balaji Medical College and Hospital, Chennai, IND; 2 Department of Radiology, Saveetha Medical College and Hospital, Saveetha Institute of Medical and Technical Sciences (SIMATS), Chennai, IND

**Keywords:** abdominal wall sinuses, chronic discharging tracts, foreign body reaction, non-absorbable sutures, prolene granuloma

## Abstract

This case report details a rare instance of multiple chronic discharging abdominal wall sinuses associated with Prolene granuloma in a 35-year-old woman following a caesarean section. Abdominal wall sinuses, though uncommon, can arise as complications after surgery, often due to foreign body reactions to materials such as non-absorbable sutures. Prolene granuloma, a rare inflammatory response to Prolene sutures, can manifest as painful masses or discharging sinuses long after surgery. In this case, the patient developed a ruptured blister at the Pfannenstiel scar, progressing to an ulcer and multiple foul-smelling sinuses. A CT scan revealed a linear hypodense track in the lower abdominal wall, suggestive of a foreign body reaction. Surgical exploration confirmed the presence of Prolene granuloma, which was successfully excised along with the sinus tracts. Histopathological analysis confirmed the diagnosis. This case highlights the need to consider Prolene granuloma in post-surgical complications, emphasizing the importance of early recognition and surgical intervention to prevent further issues and ensure symptom resolution.

## Introduction

Abdominal wall sinuses are a relatively rare but significant complication that can arise following surgical procedures, often presenting as chronic discharging tracts that may persist long after the initial surgery [1]. These sinuses can develop due to a variety of factors, including infections, foreign body reactions, poor wound healing, hematoma formation, or other postoperative complications, such as abscess formation. Among these causes, foreign body reactions to non-absorbable sutures, such as Prolene, are particularly uncommon but noteworthy.

Prolene, a non-absorbable synthetic suture material, is widely used in surgical practice due to its durability and minimal tissue reactivity. However, in rare instances, it can elicit a foreign body reaction that leads to the formation of a granuloma, known as a Prolene granuloma. This granuloma is characterized by chronic granulomatous inflammation and can manifest as painful masses or persistent discharging sinuses, often presenting months to years after the initial surgical intervention.

The pathophysiology of Prolene granuloma involves the body’s immune response to the non-absorbable suture material. When the immune system identifies the Prolene suture as a foreign entity, it triggers a chronic inflammatory response, leading to the formation of granulomas. These granulomas can result in localized inflammation, fibrosis, and the development of discharging sinuses. The clinical presentation of Prolene granuloma can be deceptive, mimicking other conditions such as abscesses, scar endometriosis, fistulae, or even recurrent tumors [2]. This makes the diagnosis challenging and necessitates a high degree of clinical suspicion, particularly in patients with a history of surgeries involving non-absorbable sutures.

This case report details a rare occurrence of multiple chronic discharging sinuses associated with Prolene granuloma in a 35-year-old female who had undergone an uncomplicated caesarean section six months earlier [3].

## Case presentation

A 35-year-old female, six months post-caesarean section, presented with a ruptured blister above the Pfannenstiel scar that progressed into a 2x1-cm ulcer, characterized by foul-smelling, seropurulent discharge and multiple 0.2x0.2-cm draining sinuses. The ulcer had sloping edges and a sloughed floor, with surrounding tenderness and induration. The patient is gravida 2, para 2, with two living children, having had one normal vaginal delivery and the recent caesarean section, with no other prior surgical history. Laboratory investigations revealed an elevated white blood cell (WBC) count of 12,500/mm³ with neutrophil predominance (80%), elevated CRP at 48 mg/L, and an increased ESR of 35 mm/hr, all indicating significant inflammation. Blood cultures were negative for systemic infection, but the wound culture grew Staphylococcus aureus, sensitive to cloxacillin and cefazolin, confirming a localized bacterial infection within the discharging sinuses.

**Figure 1:**
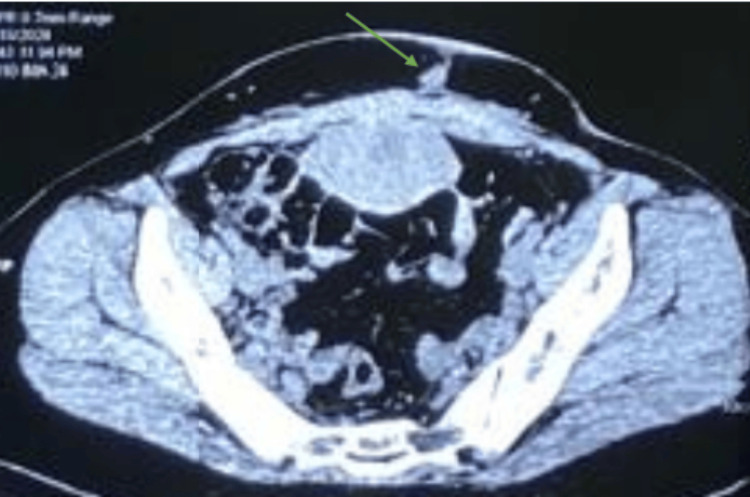
CT abdomen axial section showing a 2.3-cm linear hypodense track (green arrow) extending from the lower anterior abdominal wall to the rectus muscle.

A CT scan of the abdomen revealed a 2.3-cm linear hypodense track (green arrow) extending from the lower anterior abdominal wall to the rectus muscle, accompanied by ill-defined fat stranding (Figure 1). No abscesses or fluid collections were noted on imaging. These findings were suggestive of a foreign body reaction, likely because of Prolene sutures, leading to the suspicion of a Prolene granuloma.

**Figure 2:**
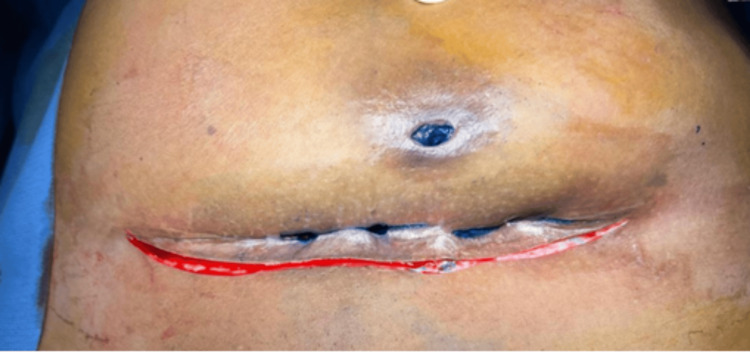
An elliptical incision made around the previous scar and methylene blue dye injected through the sinus opening.

The patient underwent surgical exploration, wherein multiple sinus tracts extending up to the anterior rectus sheath were identified (Figure 2).

**Figure 3:**
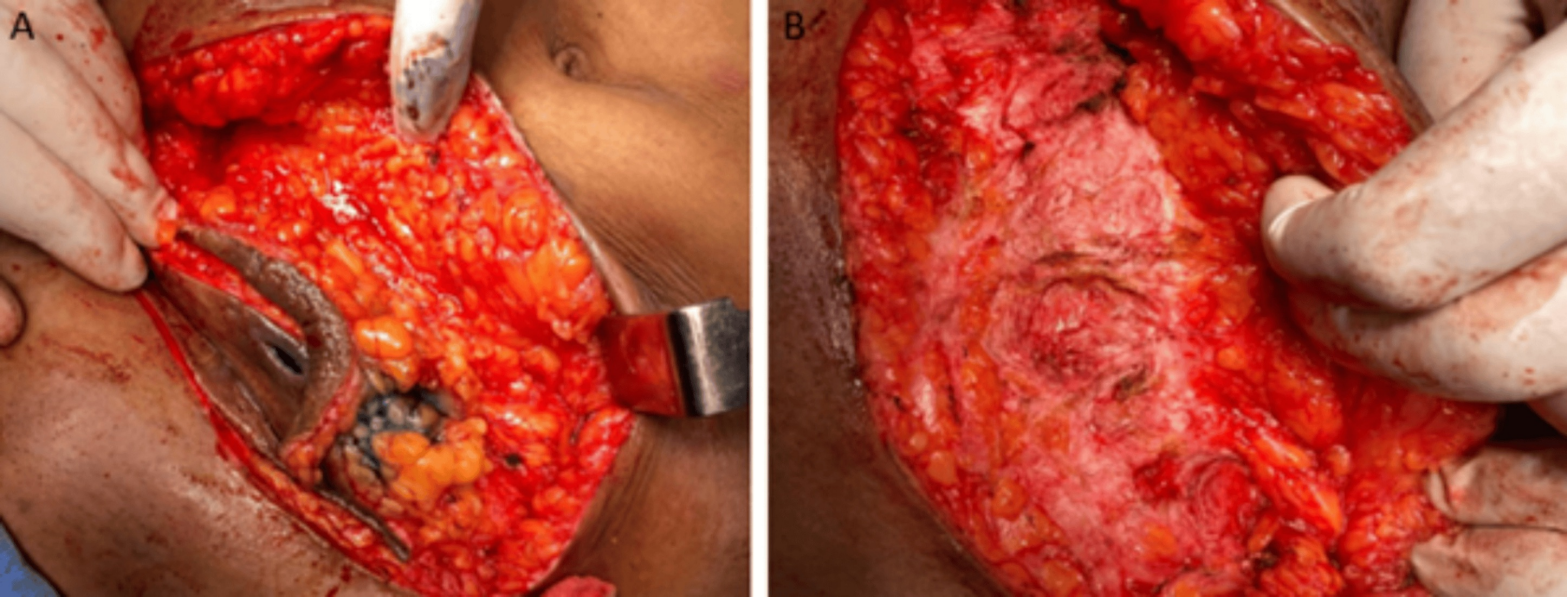
Multiple sinus tracts extending up to the anterior rectus sheath were identified.

Intraoperative dye tracking revealed Prolene granuloma along the entire suture line. The procedure involved the meticulous excision of the granuloma and associated sinus tracts, followed by removing all suture material (Figure 3A-B).

**Figure 4:**
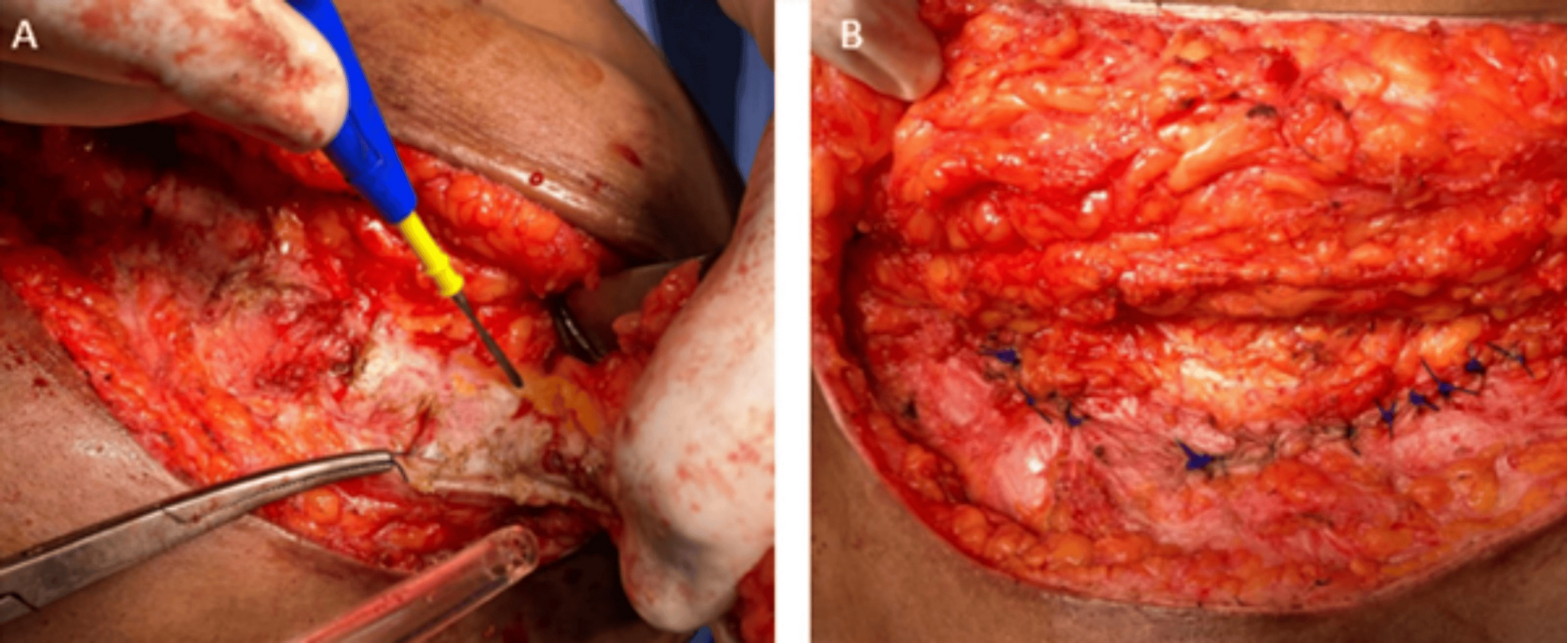
Excision of the granuloma and associated sinus tracts, followed by the removal of all suture material. After the excision, the surgical site was carefully reconstructed. (original image).

After the excision, the surgical site was carefully reconstructed to ensure proper healing and prevent recurrence (Figure 4A-B).

## Discussion

Abdominal wall sinuses are uncommon yet significant complications that can arise following various surgical procedures [4]. These sinuses often present as chronic discharging tracts and are associated with prolonged patient morbidity. The etiology of abdominal wall sinuses is multifactorial, including infections, foreign body reactions, and other postoperative complications such as inadequate wound healing. Among these, Prolene granuloma represents a rare but noteworthy cause of such complications, characterized by granulomatous inflammation in response to non-absorbable Prolene sutures. This condition is of particular interest due to its potential to significantly impact postoperative recovery and long-term patient outcomes. Understanding the etiology, clinical presentation, diagnostic modalities, and management strategies for Prolene granuloma is crucial for effective treatment and prevention of further complications.

The development of Prolene granuloma is primarily attributed to a foreign body reaction to non-absorbable suture material, specifically Prolene [5]. Prolene, a synthetic non-absorbable suture made from polypropylene, is widely used in surgical procedures due to its strength, durability, and resistance to infection. However, in some cases, the body's immune system may recognize the Prolene suture as a foreign object, triggering a localized inflammatory response. This immune response typically unfolds in two stages: Following the surgical placement of Prolene sutures, there is an immediate, non-specific tissue response to the trauma caused by the suture or needle. This response involves the infiltration of inflammatory cells such as neutrophils and macrophages, which aim to contain and neutralize any potential threats, including foreign material. Over time, if the suture material persists, a more specific inflammatory response develops, characterized by the formation of a granuloma. A granuloma is a localized nodular inflammation comprised of macrophages that have transformed into epithelioid cells, surrounded by lymphocytes and, in some cases, multinucleated giant cells. This granulomatous reaction is an attempt by the immune system to wall off and isolate the non-absorbable foreign body, which in this case is the Prolene suture. If the sheath is closed improperly with Prolene, it can create a small gap or area of incomplete closure, allowing bacteria or fluid to accumulate. This can act as a nidus for infection, leading to chronic inflammation and sinus formation at the surgical site.

The granulomatous inflammation can lead to tissue remodeling and fibrosis, further complicating the clinical picture. The persistence of the suture material within the tissue may result in chronic inflammation, leading to the formation of a sinus tract that can discharge sero-purulent material, as the body attempts to expel the foreign substance.

The clinical presentation of Prolene granuloma can vary widely depending on the severity and extent of the inflammatory response. Patients may present with symptoms ranging from mild discomfort to significant, debilitating pain [6]. The most common symptoms include pain and swelling. The affected area may be tender to palpation, with localized swelling due to the inflammatory response. Pain may be persistent or intermittent, often exacerbated by physical activity or pressure on the affected area. In many cases, a discharging sinus develops at the site of the granuloma. The discharge is typically sero-purulent and may have a foul odor, indicating the presence of secondary infection. Chronic discharge is a key feature of Prolene granuloma and often leads patients to seek medical attention. In some cases, the granuloma may present as a palpable mass at the surgical site. This mass may remain asymptomatic for months or even years, only becoming apparent when a sinus forms or when the mass enlarges significantly. In the case under discussion, the patient presented with multiple discharging sinuses and a foul-smelling, sero-purulent discharge from the surgical site. These findings were consistent with a chronic inflammatory process, likely exacerbated by secondary bacterial infection.

Accurate diagnosis of Prolene granuloma involves a combination of clinical assessment, laboratory investigations, and imaging studies. The following diagnostic tools are crucial in evaluating this condition: Inflammatory markers such as white blood cell (WBC) count, C-reactive protein (CRP), and erythrocyte sedimentation rate (ESR) are often elevated, reflecting the ongoing inflammatory process. While these markers are non-specific, they provide valuable information about the severity of inflammation. Imaging plays a pivotal role in diagnosing Prolene granuloma and assessing the extent of the condition. Ultrasound imaging is often the first-line modality used to evaluate the surgical site. It can help identify fluid collections, abscesses, and the presence of foreign bodies such as sutures. Ultrasound may reveal hypoechoic tracts consistent with sinus formation. CT imaging provides a more detailed assessment of the granuloma and associated sinus tracts. In the case discussed, a CT scan revealed a linear hypodense tract extending from the lower anterior abdominal wall to the rectus muscle, suggestive of a foreign body reaction. CT imaging is particularly useful in surgical planning, as it allows for precise localization of the granuloma and assessment of its relationship with surrounding structures. Definitive diagnosis is often confirmed through histopathological examination of the excised tissue. The presence of granulomatous inflammation surrounding the suture material, with or without associated foreign body giant cells, is diagnostic of Prolene granuloma. Histopathology also helps rule out other causes of chronic inflammation, such as infection or neoplastic processes.

The management of Prolene granuloma involves both medical and surgical approaches, with the primary goal being the removal of the foreign body and resolution of the inflammatory process. The cornerstone of treatment is the complete surgical excision of the granuloma along with any associated sinus tracts. Intraoperative findings often reveal multiple sinus tracts extending to deeper tissues. Complete removal of the Prolene suture material is essential to prevent recurrence. Failure to remove all foreign material may result in persistent or recurrent inflammation. After excision, the surgical site should be meticulously reconstructed to promote proper healing. This may involve layered closure of the wound, use of local flaps, or placement of drains to prevent fluid accumulation and infection. In cases where the granuloma is extensive, more complex reconstructive techniques may be necessary [7]. Post-excision, patients should be monitored closely for signs of infection, wound dehiscence, or recurrence of the granuloma. Antibiotic therapy may be indicated, particularly if there is evidence of secondary infection. Regular follow-up visits are important to ensure proper wound healing and to address any complications promptly. The excised tissue should be sent for histopathological examination to confirm the diagnosis and to ensure that all foreign material has been removed. Histopathology will typically show granulomatous inflammation with foreign body giant cells surrounding the Prolene suture material.

Prolene granuloma is a rare but significant complication that can arise following the use of non-absorbable sutures in surgical procedures. It is characterized by a foreign body reaction leading to chronic inflammation, granuloma formation, and, in some cases, the development of discharging sinuses. Early recognition and appropriate management are crucial for preventing chronic morbidity associated with this condition. Surgical excision of the granuloma and complete removal of the suture material are the mainstays of treatment [8]. Understanding the etiology, clinical presentation, and diagnostic modalities is essential for the effective management and prevention of further complications in patients with Prolene granuloma.

## Conclusions

In conclusion, multiple abdominal wall chronic discharging sinuses with Prolene granuloma represent a rare but significant postoperative complication associated with the use of non-absorbable sutures. This case underscores the importance of considering Prolene granuloma in the differential diagnosis of persistent sinuses following surgical procedures. Accurate diagnosis, primarily through imaging and histopathological examination, is crucial for guiding appropriate surgical management. The successful treatment of such cases typically involves the complete excision of the granuloma and affected sinus tracts, along with the removal of the foreign suture material, to prevent recurrence and ensure optimal patient outcomes.
